# Community Level Youth-Led Interventions to Improve Maternal-Neonatal Outcomes in Low- and Middle-Income Countries: Protocol for a Systematic Review

**DOI:** 10.1155/2022/9580986

**Published:** 2022-05-27

**Authors:** Tonya MacDonald, Avonae Gentles, Rachel Liu, Maya Stevens-Uninsky, Naharin Sultana Anni, Nadia Rehman, Elizabeth K. Darling, Saara Greene, Sandra Moll, Lawrence Mbuagbaw

**Affiliations:** ^1^Department of Health Research Methods, Evidence and Impact, McMaster University, Hamilton, ON, Canada; ^2^Faculty of Health, Health Sciences Program, University of Waterloo, Waterloo, ON, Canada; ^3^Department of Global Health, McMaster University, Hamilton, ON, Canada; ^4^McMaster Midwifery Research Centre, McMaster University, Hamilton, ON, Canada; ^5^Department of Obstetrics and Gynecology, McMaster University, Hamilton, ON, Canada; ^6^Faculty of Social Sciences, School of Social Work, McMaster University, Hamilton, ON, Canada; ^7^School of Rehabilitation Science, McMaster University, Hamilton, ON, Canada; ^8^Centre for the Development of Best Practices in Health, Yaoundé Central Hospital, Yaoundé, Cameroon; ^9^Biostatistics Unit/The Research Institute, St Joseph's Healthcare-Hamilton, ON, Canada; ^10^Division of Epidemiology and Biostatistics, Department of Global Health, Stellenbosch University, South Africa

## Abstract

**Introduction:**

On a global scale, women and childbearing people and neonates continue to die from preventable causes related to pregnancy or childbirth. Sustained and accelerated efforts are critical to improve maternal and neonatal health and well-being. Globally, youth are a growing population and have strength in their numbers. Youth are critical, key drivers of change in their communities. Young people hold the potential to affect positive change, and their meaningful engagement is important to improving maternal health and well-being in low- and middle-income countries.

**Objectives:**

To assess the effects of community level youth-led interventions for improving maternal-neonatal health and well-being compared with no interventions or another intervention.

**Methods:**

We will undertake a literature search that is comprehensive, complete, and exhaustive. This will include databases such as MEDLINE, EMBASE, and the Cochrane Library, as well as a grey literature search. In our systematic review, we will include experimental studies evaluating maternal-neonatal health and well-being associated with or because of the implementation of community level youth-led interventions. Participants will include women and childbearing people (of any age) during antepartum, intrapartum, and postpartum periods (up to 42 days postpartum). We will examine all interventions addressing and targeting maternal-neonatal health and well-being that are youth-led and community-based and aimed at the members of the community. Our comparators will be no intervention or another intervention. Our primary outcomes are maternal deaths and neonatal deaths. Our review will include only studies in low- and middle-income countries conducted in urban or rural areas. *Ethics and Dissemination*. Ethics approval is not required as we will use secondary data that is publicly available. There are no active participants in our study. We will involve key stakeholders and experts in maternal-neonatal health regarding dissemination and knowledge mobilization strategies. Our findings will be disseminated as an open access publication, be presented publicly, and defended as part of a doctoral thesis. This trial is registered with CRD42021288798.

## 1. Introduction

Everyday globally hundreds of women and childbearing people and thousands of neonates (newborns) die from preventable causes related to pregnancy or childbirth. In our communities and within countries and across nations, global maternal and neonatal survival are inequitable and represent critical challenges that must be overcome. This is a matter of social justice not only for the health and well-being of mothers and childbearing people and their babies but also for the health (and wealth) and well-being of their communities, countries, and nations.

Maternal and neonatal death are embedded within a context of inequity and fueled by a lack of human, financial, and infrastructural resources especially in countries with the highest maternal mortality rates (MMRs) and neonatal mortality rates (NMRs). In low- and middle-income countries (LMICs) and least developed countries, i.e., the “global south”, there are extremes of inequity in survival. Currently, the MMR point estimate for Europe and Northern America is 12 maternal deaths per 100,000 live births; for subregions of Asia and Western Africa, there is a MMR point estimate range of 69 to 151 [1]. The MMR point estimate is highest for the least developed countries at 415 maternal deaths per 100,000 live births and remains overall highest in the region of Sub-Saharan Africa with an MMR point estimate as high as 542 maternal deaths for 100,000 live births [1]. Globally, in 2019, the average rate of neonatal deaths was 17 deaths per 1,000 live births [2]. Like maternal death, there is also widespread regional disparities in neonatal and under-five chances of survival, with the region of Sub-Saharan Africa having the highest under-five mortality rate in the world [2].

Sustained and accelerated efforts in communities, across jurisdictions, nations, and countries and on the global stage are critical to improve maternal and neonatal health and well-being. These efforts must be aimed at reducing the number of women and childbearing people from dying during pregnancy, in labour and birth, and in the 42 days following birth or termination of pregnancy (i.e., maternal mortality) and at reducing the number of neonates dying in the first 28 days after their birth. In the post-Millennium Development Goals (MDGs) era and renewed by the United Nation's Sustainable Development Goals (SDGs), nations are committed to working to end preventable maternal mortality and neonatal death globally and ultimately. With the goal of eliminating extremes of inequity in global maternal and neonatal survival by 2030, we aim for an average global target MMR of less than 70 maternal deaths per 100,000 live births, and a supplementary national target that will have no country with an MMR greater than 140 deaths per 100,000 live births [3]. In terms of neonatal survival, we are aiming for an average global target of fewer than 25 neonatal deaths per 1,000 live births [2].

While a lack of financial or infrastructural resources may be a common denominator for countries still working to reduce their MMR and NMR, also common to many of these same LMICs is their proportionately high youth populations. Youth—defined by the United Nations (UN)—as people aged 15-24 years [4] have strength in their numbers. Investment in youth, through greater participation and opportunities for meaningful engagement involved in policy development and decision-making at local, national, regional, and international levels [5], offers the opportunity to unleash the human potential of this “Sustainable Development Goals Generation” in order to transform our world [6]. Could youth, an often marginalized, disregarded, and voiceless group [7, 8], be an untapped, available, and willing resource to improve maternal health and well-being in their communities?

Young people have often been overlooked in terms of their meaningful engagement and active role in their communities. However, globally, there are many examples of youth-focussed and youth-led interventions in health, governance and democracy, economic development, and education [9]. For example in Sub-Saharan Africa, a Randomized Controlled Trial (RCT) in Zambia assessed the impact of youth-led strategies in clinic-based programs aimed at improving viral suppression and reduce stigma among HIV-positive youth [10]. This study concluded that their well-trained and paid adolescents and young adults implemented the key intervention successfully, leading to an improvement in HIV-related outcomes [10]. Another example is a youth-led education intervention in rural Pakistan. In this cluster RCT, trained female youth (age 19-24 years) delivered an early childhood care and education program to young children (age 3.5-6.5 years) [11]. This cross-generational intervention was led by youth working directly with children in their communities and was shown to be effective in supporting early childhood development and young children's school readiness [11]. This is an example of youth as transformative change agents where both the young and the community at large are beneficiaries.

From another perspective, a study in southern Africa examined reasons for the failure of HIV and intimate partner violence interventions for young women [12] to find that failure to focus on broader social and structural contexts and the absence of meaningful involvement of youth in designing interventions created barriers and impeded the success of the interventions [12]. This underscores the potential role of youth in health outcomes.

In youth-led interventions, proponents not only regard youth as important agents of social awareness and transformation but cite “higher levels of creativity and energy among youth and a higher potential to introduce innovations compared to adults” [11]. Youth-led interventions, such as those in health, education, governance and democracy, and economic development, are aimed at benefiting the community while engaging the community's future leaders. Meaningful youth involvement has the potential to broaden social and structural contexts that strengthen health and well-being. With specific regard to maternal-neonatal health, youth-led interventions hold the potential to promote positive health behaviours, strengthen community understanding of barriers to health services access, support educational activities related to reproductive and sexual health, and advocate for health equity among marginalized populations such as women, girls, youth, and LGBTQ2 folks, for example.

Our systematic review seeks to examine youth-led interventions in communities, where youth are involved in leading or delivering all or part of interventions. These interventions need not be youth-designed or youth-initiated. However, youth-led implies the bona fide involvement of youth-engaged as consultants or trainees or mentees, in study conception or design, in recruitment, administration of intervention, data collection, analysis, or knowledge translation. The engagement of youth in the intervention also implies a benefit to youth in terms of fostering skills development (e.g., leadership, research, and mentorship), socioeconomic opportunity, and personal growth.

Youth are a growing population, globally and particularly in LMICs. It is critical to engage youth who understand the broad social and structural contexts of their communities so they can be key drivers of change in their communities. Young people hold the potential to affect positive change, and their meaningful engagement may be important to improving maternal health and well-being in LMICs.

### 1.1. Objectives

Our objective is to assess the effects of community level youth-led interventions for improving maternal-neonatal health and well-being compared with no interventions or another intervention.

## 2. Methods

This protocol is reported according to the PRISMA-P (Preferred Reporting Items for Systematic Reviews and Meta-analyses for systematic review protocols) guidelines [13].

### 2.1. Eligibility Criteria

#### 2.1.1. Study Designs

We will include experimental studies evaluating maternal-neonatal health and well-being associated with or because of the implementation of community level youth-led interventions. We will include randomized control trials (RCTs), quasirandomized trials, and cluster randomized trials (CRTs).

#### 2.1.2. Participants

We will include studies of maternal health outcomes of women and childbearing people at different maternal periods, who have received a youth-led intervention, and where there was evaluation of maternal or neonatal health and well-being. This will include women and childbearing people (of any age) during antepartum, intrapartum, and postpartum periods (up to 42 days postpartum). The postpartum period, also called the postnatal period, is defined by the World Health Organization (WHO) as the first six weeks after childbirth [14]. It is within this critical period that most maternal deaths occur but also presents a vital opportunity to improve both maternal and neonatal health and well-being [14].

#### 2.1.3. Interventions

We will examine all interventions addressing and targeting maternal-neonatal health and well-being that are youth-led and community-based and aimed at members of the community. These may include clinical, educational, behavioural, or policy interventions or other types of interventions that may be nonspecific, multipronged, preventative, or therapeutic in nature.

Our review will include studies with single components, i.e., consisting of a single intervention (e.g., educational materials) or multiple components (e.g., educational material, education sessions, and home visits) of any duration.

Interventions must be youth-led, where youth are people 15-24 years old [4]. We will include studies where youth were involved in leading or delivering all or part of the interventions. It is not necessary that interventions be youth-designed or initiated. We will include studies with any duration of follow-up and with no restrictions on timing.

#### 2.1.4. Comparators

Our comparators will be no intervention, or another intervention.

#### 2.1.5. Outcomes

Our primary outcomes are as follows:

1. Maternal deaths (i.e. pregnancy-related deaths): proportion of pregnant women/people who die during pregnancy or within 42 days of termination of pregnancy, irrespective of cause (obstetric, nonobstetric, accidental, or incidental) [1]
2. Neonatal deaths: proportion of newborn infants who die in the first 28 days after birth [15]

Our secondary outcomes are as follows:

1. Antenatal Care Coverage (ANC): proportion of pregnant women/people who attend at least four antenatal care visits during pregnancy [16]
2. Births attended by skilled health personnel: proportion of pregnant women/people giving birth in health facilities
3. Adolescent birth rate: proportion of young women/people aged 15-19 years giving birth
4. Stillbirth: proportion of infants who die after 28 weeks of pregnancy, or before or during birth [17]
5. Postpartum Hemorrhage (PPH): proportion of women/people who suffered from blood loss of 500 ml or more within 24 hours after birth [18]
6. Maternal near-miss: proportion of women/people “who nearly died but survived a complication that occurred during pregnancy, childbirth or within 42 days of termination of pregnancy” [19]

#### 2.1.6. Study Settings

Our review will include only studies in LMICs, conducted in urban or rural areas. We will use the World Bank list of countries (2019), classified as low-income, lower-middle-income, or upper-middle-income economies (i.e., countries) [20]. In cross-country studies, we will consider only data from the LMIC.

### 2.2. Information Sources and Search Strategy

We will construct a literature search strategy that is comprehensive, complete, and exhaustive. We will seek the guidance of Health Sciences Librarians with expertise in systematic review searching. Our strategy will be peer reviewed by the authors of the systematic review as well as an information specialist, with reference to the guideline for Peer Review of Electronic Search Strategies (PRESS) [21].

We will use strategies customized to each database and their controlled vocabularies for medical subject headings (MeSH), key word, and truncation search structures. The search will be based on three main concepts: youth-led interventions, LMIC/low-resource settings, maternal-neonatal health, and well-being. Cochrane's Effective Practice and Organisation of Care (EPOC) LMIC filters will be used for a list of low-to-middle-income countries [20]. We will search clinical trial registries for ongoing or recently completed clinical trials. We will include unpublished material or abstracts where appropriate but will filter out and exclude other records such as editorials and commentaries. Our search will be updated toward the end of the review to ensure that it is up to date. A draft search strategy for Medical Literature Analysis and Retrieval System Online (MEDLINE) is shown as an example (supplementary file 1).

We will search the following electronic databases:

1. MEDLINE (Ovid interface, 1946 to present)
2. EMBASE (Ovid interface, 1974 to present)
3. CINAHL (EBSCO interface, 1981 to present)
4. Global Health (Ovid interface, 1910 to present)
5. Web of Science (1976 to present)
6. Cochrane Central Register of Controlled Trials
7. World Health Organization (WHO) International Clinical Trials Registry Platform (ICTRP) and Clinicaltrials.gov for ongoing trials
8. LILACS (Latin American and Caribbean Health Sciences literature)
9. United Nations and relevant reports from the publication websites of United Nations Population Fund (UNFPA), United Nations Children's Fund (UNICEF), WHO and Centers for Disease Control and Prevention (CDC)

There will be no date or language restrictions applied. We will search grey literature sources such as the Global Health Library, World Bank, Emergency Nutrition Network, ALNAP Overseas Development Institute, and Eldis. To further supplement our search, we will also examine major conference websites for relevant abstracts from conference proceedings. Authors of relevant articles will be contacted by email (to maximum of three attempts) to locate other relevant published or unpublished studies. Key stakeholders and experts in maternal-neonatal health will be contacted by email or phone for additional sources of literature. Finally, the reference lists of all relevant studies will be checked for additional studies for inclusion that may have been missed.

### 2.3. Study Records

#### 2.3.1. Data Management

We will use EndNote X9, a bibliographic software [22], to download search results and remove duplicates in preparation of the screening process. We will use DistillerSR, a web-based software management program designed for systematic reviews, to help facilitate data management and collaboration among systematic review team members during the data extraction process [23]. Using our inclusion and exclusion criteria, we will develop and test screening questions and data collection forms prior to the formal screening process.

#### 2.3.2. Selection Process

The selection of study articles for inclusion will occur in two stages. First, the reviewers (TM, RL, MSU, NSA, and NR) will independently scrutinize the titles and abstracts of all retrieved studies, to determine whether they meet the inclusion criteria. We will exclude those that are not relevant to our objectives; that is, those experimental studies do not concern youth-led interventions to improve maternal-neonatal health and well-being in LMICs. In the second stage, the reviewers (three separate teams) will independently and in duplicate review the full-text versions of articles deemed to meet the criteria to make a final decision about study inclusion or exclusion. Irrelevant articles will be excluded. At both stages of study selection, the reviewers will resolve any disagreement through discussion or by involving a third-party adjudicator with systematic review expertise (e.g., LM). We will prepare a PRISMA (Preferred Reporting Items for Systematic Reviews and Meta-analyses) study flow diagram [24] [25] showing in a sequential manner the number of study articles searched, screened, identified, included, and excluded along with the reasons for rejected studies.

#### 2.3.3. Data Collection/Extraction Process

We will design and pilot test a data extraction form. The standardized form will include bibliographic information, location, setting, study design, methodology, intervention details, and all reported outcomes. TM and NR will extract data independently and in duplicate, by appraising the full text of the included studies, using the agreed form and resolving discrepancies through discussion. Both reviewers will verify the extracted data. As needed, another review author (e.g., LM) will be consulted if there is no consensus. Study investigators will be contacted to resolve any uncertainties. If there are multiple reports as separate studies, the independent reviewers will discuss any apparent duplicate, overlapping, or companion studies that come to light. These will involve a third-party adjudicator regarding a planned approach for resolving inconsistencies across reports. We will enter data into Review Manager software [26] and check for accuracy.

Agreement on screening at both stages will be estimated using the kappa (*κ*) statistic. The strength of agreement will be interpreted as follows: poor (*κ* ≤ 0.2), fair (0.21 ≤ *κ* ≤ 0.4), moderate (0.41 ≤ *κ* ≤ 0.6), substantial (0.61 ≤ *κ* ≤ 0.8), or almost perfect (*κ* > 0.8) [27].

### 2.4. Data Items

We will extract the details of the type of intervention, type of control used, participant characteristics, types of outcome measures extracted, trial design, trial size, duration of follow-up, type and source of financial support, and publication status from trial reports.

### 2.5. Risk of Bias of Individual Studies

TM and NR will individually and independently assess for the risk of bias for each included study. The reviewers will resolve any disagreement through discussion and third-party adjudicator involvement as necessary. We will assess risk of bias using the Cochrane risk-of-bias tool for randomized trials (RoB 2) [28] and Cochrane's RoB 2 test version tool for cluster-randomized trials [29]. Our screening/extraction tool for risk of bias will integrate the RoB 2 five domains and signaling questions as dimensions of bias assessment. Our tool will include these five domains: randomization process, intended interventions, outcome data, measurement of the outcome, and selection of the reported result. For each domain, a study can be judged to be at “low risk of bias,” having “some concerns,” or “high risk of bias” [28]. The risk of bias tables will be filled independently by the two reviewers.

Since it is likely our systematic review will include cluster-randomized trials, we will also consider additional sources of bias [30] such as the following:

- Recruitment bias: of individuals recruited into the trail after clusters were formed
- Baseline imbalances: due to small numbers of clusters
- Attrition rate of entire clusters
- Methods of analysis related to correlation between members of the same cluster
- Comparability with individually randomized trials [31]

### 2.6. Data Synthesis

If studies are sufficiently similar to pool, we will conduct a meta-analysis of outcomes using a random effects model. Statistical analysis will be done using the Review Manager software [26].

#### 2.6.1. Measures of Treatment Effect

For studies where data are appropriate for synthesis of dichotomous outcomes, we will determine treatment effect by using risk ratio (RR) with 95% confidence interval (CI); for continuous outcomes, we will use weighted mean differences or standardized mean differences (95% CI) and present nonquantitative data descriptively.

#### 2.6.2. Unit of Analysis Issues

We plan to include cluster-randomized trials in the analyses along with individually randomized trials. We will adjust for the sample sizes of included cluster-randomized trials by following Cochrane-described methods of using an estimate of the interclass correlation co-efficient (ICC) [30].

#### 2.6.3. Other Unit of Analysis Issues

For included studies that may have more than one intervention arm, we will only use data from eligible arms in pair-wise comparisons.

#### 2.6.4. Dealing with Missing Data

For all studies, we will use the intention-to-treat analysis as reported. Where there are large amounts of missing data, we will conduct a sensitivity analysis to determine the impact of missing data.

#### 2.6.5. Assessment of Heterogeneity

We will first assess all included studies for clinical heterogeneity, i.e., sufficient similarity across studies in terms of participants, interventions, comparisons, and outcomes. If they are sufficiently similar, we will pool them in a meta-analysis and evaluate statistical heterogeneity using chi^2^, *I*^2^, *T*^2^, and statistics. We will use the chi^2^ test with a significance level of alpha = 0.10 and the *I*^2^ test to evaluate the extent of inconsistency of studies' results.

#### 2.6.6. Subgroup Analysis and Investigation of Clinical Heterogeneity

We will use subgroup analysis to investigate unexplained heterogeneity. Possible sources of heterogeneity and potential subgroups for analysis may include age, duration of intervention, male versus female youth-led interventions, and urban versus rural setting.

1. Age: due to stigma around unplanned pregnancy or adolescent pregnancy, we hypothesize that pregnant youth will have lower rates of accessing at least four antenatal care visits compared to pregnant adults.
2. Duration of intervention: we hypothesize that interventions of longer duration will have lower rates of stillbirth, PPH, and maternal near-misses.
3. Male versus female youth-led interventions: we hypothesize that female-led interventions will have lower rates of adolescent birth over male-led interventions.
4. Rural versus urban setting: we hypothesize that participants living in more urban areas will have higher skilled birth attendance and fewer maternal and neonatal deaths, over participants living more rurally.

#### 2.6.7. Assessment of Reporting Biases

If we have 10 or more studies for our primary outcomes, we will assess publication bias by checking for asymmetry of the funnel plot. [32]. We will also conduct Egger's test for small study-effects [27].

If data cannot be pooled from our included studies, we will include a narrative description that synthesizes the range of impact the different interventions had on maternal and neonatal health and well-being. Synthesis of the data will consider the following questions:

- How were maternal-neonatal health and well-being evaluated?
- How were youth engaged in the intervention?
- For which interventions were there reported positive or negative maternal-neonatal effects?
- What were the quantitative measures of effects used for maternal-neonatal outcomes?
- How were study results presented regarding impact on maternal and neonatal health and well-being?
- How can the results of the different studies be combined?

### 2.7. Certainty of Evidence

We will assess and summarize the quality of the overall retrieved body of evidence generated through our systematic review using GRADE (Grading of Recommendations, Assessment, Development and Evaluation) tool [33]. The quality of evidence across studies will be adjudicated as high, moderate, low, or very low. Strength of evidence will be evaluated across the domains of risk of bias, consistency, directness, precision, and publication bias. We will assess factors that decrease the quality of evidence such as limitations in design, indirectness of evidence, inconsistency or imprecision of results, unexplained heterogeneity, or high probability of publication bias [34]. For qualitative data, we will apply the GRADE-CERQual (Confidence in the Evidence from Reviews of Qualitative research) for appraisal and to summarize evidence in a succinct, transparent, and informative way [35].

## 3. Discussion

Our systematic review will generate evidence on the impact of youth-led interventions in low-to-middle-income countries applied to people of childbearing age. To our knowledge, this will be the first systematic review on effects of community level youth-led interventions on maternal and neonatal health and well-being. It is expected that this study will identify knowledge gaps and potential solutions regarding youth-led interventions in LMICs. Results from our study could be considered in future community planning and policy developments that are aimed at improving maternal-neonatal health and well-being and at including youth engagement in maternal-neonatal health initiatives in LMICs. One potential study limitation is that heterogeneity across the included studies pertaining to participant characteristics, types of intervention, comparators, follow-up period, etc. may preclude statistical pooling.

### 3.1. Ethics and Dissemination

Since we will use secondary publicly available data, ethics approval is not required. As there will be no active participants in our study's design, conduct, analysis, or dissemination, consent for publication and dissemination of findings are not required. We will involve key stakeholders and experts in maternal-neonatal health for any recommendations regarding dissemination and knowledge mobilization strategies. Our findings will be disseminated as an open access publication to increase equitable access by researchers, community stakeholders, maternal-neonatal providers, and policy and decision makers. Our findings will also be presented publicly and defended as part of a doctoral thesis.

## Data Availability

All datasets on which the conclusions of the paper rely are publicly available to readers. A list of these will be either presented in the main manuscript of our systematic review or as additional supplementary information files whenever possible.

## Abbreviations

ANC: Antenatal Care Coverage
CDC: Centers for Disease Control and Prevention
CI: Confidence interval
CINAHL: Cumulative Index to Nursing and Allied Health Literature
CRT: Cluster randomized trial
EPOC: Effective Practice and Organisation of Care
EMBASE: Excerpta Medica Database
GRADE: Grading of Recommendations, Assessment, Development and Evaluation
GRADE-CERQual: Confidence in the Evidence from Reviews of Qualitative research
ICTRP: International Clinical Trials Registry Platform
LILACS: Latin American and Caribbean Health Sciences literature
LMIC: Low- and Middle-Income Country
MMR: Maternal Mortality Ratio
MeSH: Medical subject heading
MEDLINE: Medical Literature Analysis and Retrieval System Online
MDG: Millennium Development Goal
NMR: Neonatal Mortality Ratio
PRESS: Peer Review of Electronic Search Strategies
PRISMA: Preferred Reporting Items for Systematic Reviews and Meta-analyses
PRISMA-P: Preferred Reporting Items for Systematic Reviews and Meta-analyses for systematic review protocols
PPH: Postpartum Hemorrhage
RCT: Randomized Controlled Trial
RoB 2: Risk-of-bias tool for randomized trials
SDG: Sustainable Development Goal
UN: United Nations
UNFPA: United Nations Population Fund
UNICEF: United Nations Children's Fund
WHO: World Health Organization.

## Supplementary Materials

Supplementary Materials Supplementary file 1 is a draft search strategy for Medical Literature Analysis and Retrieval System Online (MEDLINE).
